# Effects of Depilation-Induced Skin Pigmentation and Diet-Induced Fluorescence on In Vivo Fluorescence Imaging

**DOI:** 10.1155/2017/7659242

**Published:** 2017-06-19

**Authors:** Sunkuk Kwon, Eva M. Sevick-Muraca

**Affiliations:** Center for Molecular Imaging, The Brown Foundation Institute of Molecular Medicine, The University of Texas Health Science Center, Houston, TX 77030, USA

## Abstract

Near-infrared fluorescence imaging (NIRFI) and far-red fluorescence imaging (FRFI) were used to investigate effects of depilation-induced skin pigmentation and diet-induced background fluorescence on fluorescent signal amplitude and lymphatic contraction frequency in C57BL6 mice. Far-red fluorescent signal amplitude, but not frequency, was affected by diet-induced fluorescence, which was removed by feeding the mice an alfalfa-free diet, and skin pigmentation further impacted the amplitude measurement. NIRFI showed minimal background fluorescence; however, skin pigmentation reduced the amplitude of fluorescent signal changes. Therefore, these effects should be taken into account when imaging mice with different states of skin pigmentation and diet-induced background fluorescence in vivo.

## 1. Introduction

There are several challenges to applying in vivo fluorescence imaging, since tissue absorption and scattering can significantly impact the measured spectrum of the fluorophore. For in vivo fluorescence imaging in rodents, animals typically need to be clipped and depilated to remove residual hair before imaging, in order to reduce hair scattering effects and thus to improve collection of fluorescent signals. However, it has been shown that the use of chemical depilation in C57BL6 mice results in skin pigmentation due to disruption of normal hair growth. Hair follicles undergo a cycle of growing (anagen), regressing (catagen), and resting (telogen) phases [1, 2]. In a resting telogen phase, the skin of the mice is generally unpigmented light pink [3]. However, pigmentation occurs in anagen phase due to melanin pigment produced actively in the hair follicles, followed by a regression (catagen) phase where hair growth stops [3]. Thus, chemical depilation is induced to enter anagen in C57BL6 mice in which hair follicles are in telogen [3]. As a result, in vivo longitudinal fluorescence (and bioluminescence) imaging in the same C57BL6 mouse over time, or among different mice with varying degrees of skin pigmentation, may cause the qualitative and quantitative imaging to be compromised due to fluorescent signal attenuation in the target region, resulting from changes in melanin concentration.

In addition to skin pigmentation, background fluorescence is known to provide a limitation for in vivo fluorescence imaging. It has been demonstrated that, after being excited by about 660 nm excitation light, mice fed a normal animal diet show strong diet-induced fluorescence in the gastrointestinal (GI) track in the abdominal region, since chlorophyll from the alfalfa in commonly used laboratory rodent diets fluoresces between 665 and 750 nm [4]. Therefore, mice were fed with alfalfa-free diet for far-red fluorescence imaging (FRFI) using red-excitable fluorophores, such as Cy5.5 and Alexa-680, or fluorescent proteins to reduce unwanted background fluorescent signals [4, 5].

Recently, we and others have developed fluorescence lymphatic imaging using red-excitable dyes (e.g., IRDye680) or near-infrared fluorophores (e.g., indocyanine green (ICG)) [6–12]. From in vivo imaging, lymphatic function measurement, such as lymphatic contraction frequency and fluorescent signal amplitude, has been quantified. However, it has not been reported about how those measurements are affected by changes in skin coloration and background fluorescence resulting from alfalfa-containing animal diet. In the present work, we longitudinally imaged and characterized dermal lymphatics using multiwavelength fluorescence imaging with injection of a mixture of Alexa-680-bovine serum albumin (BSA) and ICG in C57BL6 mice, which were fed with regular or alfalfa-free diet, in response to changes of skin pigmentation resulting from chemical depilation.

## 2. Materials and Methods

### 2.1. Animals

Six-to-eight-week-old female C57BL6 mice (Charles River Laboratories, Inc., Wilmington, MA) were maintained in a pathogen-free mouse colony at the Brown Institute of Molecular Medicine, University of Texas Health Science Center at Houston (UTHSC-H), which is accredited by the Association for the Assessment and Accreditation of Laboratory Animal Care. Mice were fed a regular or alfalfa-free diet (AIN-93G; OpenSource Diets, New Brunswick, NJ, USA). All animal experimentation was performed after approval by the Institutional Animal Care and Use Committee.

### 2.2. Fluorescence Imaging

Mice were clipped and a depilatory agent (Nair, Church & Dwight Co., Inc.) was used to remove residual hair 24 hr before photographic imaging, every week, for 6 weeks. During imaging, animals were placed under isoflurane anesthesia and maintained on a 37°C heating pad. Fluorescence imaging was conducted at week 1 and week 3. A volume of 10 ul of a mixture of Alexa-680-BSA (25 *µ*g) and ICG (2.5 *µ*g) was injected to the base of the tail using a 31-gauge needle. The injection site was covered by black tape to prevent oversaturation of the camera. NIR and far-red fluorescent images were acquired for 5 mins at 5 mins after injection of the mixture. A laser diode (500 mW, 785 nm for ICG excitation; 500 mW, 660-nm for Alexa-680 excitation) provided excitation light, which uniformly illuminated the whole body of the mouse. To reject backscattered and reflected excitation light, a holographic notch-plus band rejection filter (785 nm and 660 nm center wavelength for NIRFI and FRFI, resp.) and a bandpass filter (830 nm and 710 nm center wavelength for NIRFI and FRFI, resp.) were positioned prior to the 50-mm lens. Therefore, reemitted fluorescence light was detected by an electron multiplying charge-coupled device (EMCCD) camera (Photometrics).

### 2.3. Data Analysis

The data were analyzed with ImageJ (National Institutes of Health, Bethesda, MD). The skin pigmentation levels were measured as mean gray values [3] by selecting regions of interest (ROIs) over the internodal collecting lymphatic vessels in the left lateral side of mice in the photographic images acquired using a stereomicroscope. To reveal contractile activity, the same size of fixed ROIs in fluorescent internodal lymphatic vessels connecting from the inguinal lymph node (ILN) to the axillary LN (ALN) (1.5 cm away from the ILN) was selected. The ROIs were selected at the same location in all mice. The averaged fluorescence intensity within each ROI in each fluorescence image was obtained. The profiles were normalized to maximum intensity and plotted as a function of imaging time. The number of “pulses” is an indication of lymphatic contractile activity and termed “contractions.” In addition, a ROI over the stomach region was selected to measure diet-induced background fluorescence.

Data were presented as average values ± standard error of the mean (SEM). Statistical analysis was performed with GraphPad Prism 5 (GraphPad Software, Inc.). The data were tested for normality using a D'Agostino and Pearson Omnibus normality test. For non-Gaussian distributed data, one-way ANOVA Friedman's test with Dunn's multiple comparison test was used for multiple comparisons, or Mann–Whitney or Wilcoxon tests were used to compare two unpaired or paired groups, respectively. In addition, comparisons between two groups were made with Student* t*-test for paired or unpaired parametric data. Statistical significance is based on *P* values, with *P* < 0.05 considered a significant difference.

## 3. Results

**Figure 1:**
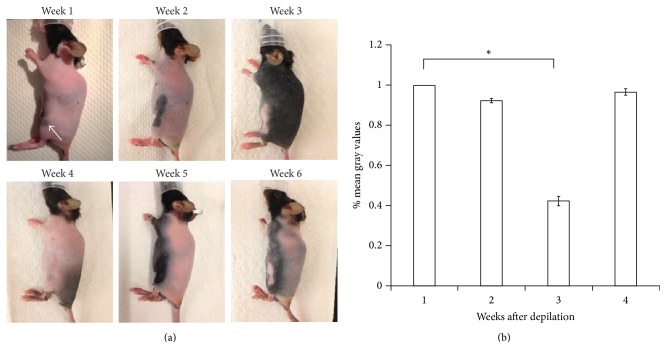
(a) Representative photographic images of 3 mice from weeks 1 to 6 after depilation. (b) Percent mean gray values of weeks 1–4 (*n* = 4; one-way ANOVA Friedman's test with Dunn's multiple comparison test). Data are shown as mean ± SEM. Arrow, pigmented regions at week 1. ^*∗*^*P* < 0.0001.

Mice were depilated every week and photographed a day later. As shown in Figure 1, C57BL6 mice after the first depilation (week 1) showed pink skin in most of the animal body, although some areas were pigmented (arrow in Figure 1). The visual observation from the photographic images demonstrated increased pigmentation in mouse skin up to 3 weeks after depilation, when skin pigmentation was greatest, and a return to baseline levels in week 4. Such cycling changes last for about 4 weeks before skin pigmentation started again. We did not observe temporal changes in skin pigmentation on the whole body (Figure 1). The mean gray values, used to measure the skin pigmentation level, as shown in Figure 1(b), were significantly reduced at week 3 as compared to week 1 (*P* < 0.001), in agreement with the visual observation.

**Figure 2:**
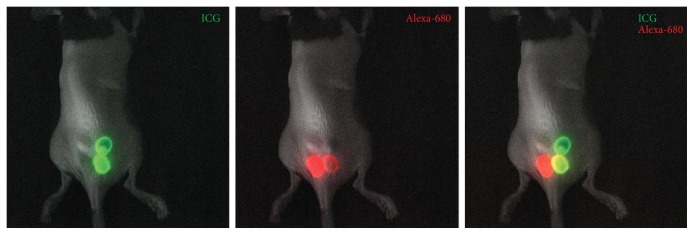
Merged images of white light images with ICG and Alexa-680 fluorescent images after injection of each dye and the mixture to the back of the skin of mice fed an alfalfa-free diet.

Next, we intradermally injected two different imaging agents with distinct excitation/emission wavelengths, first separately and then a mixture of them, into the skin of the back of mice to investigate whether there are any overlapping fluorescent emission signals. As shown in Figure 2, we detected distinct ICG and Alexa-680 fluorescence in the injection sites of each separately injected dye, as well as the mixture, in mice at week 1 after depilation.

**Figure 3:**
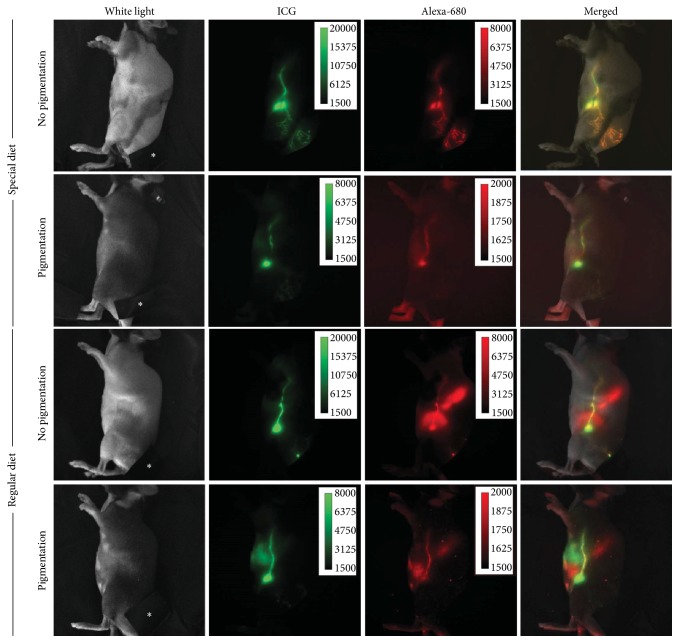
Representative white light, fluorescent, and merged images 5–10 mins after i.d. injection of 10 ul of a mixture of ICG and Alexa-680-BSA to the base of the tail in mice with or without skin pigmentation fed a regular or special diet. The injection site was covered by back tape to prevent oversaturation of the camera (asterisk).

Then we injected a mixture of imaging agents into the base of the tail and performed dynamic fluorescence lymphatic imaging to monitor attenuation of fluorescent emission signals due to skin pigmentation. All mice showed lymphatic drainage from the base of the tail to the ILNs and finally to the ALNs via the internodal collecting lymphatic vessels. We observed reduced fluorescent emission in pigmented mice (Figure 3; signal calibration bar). In groups of mice fed an alfalfa-free diet, we observed no or minimal background in the gastrointestinal track (GIT) (abdominal region) of mice with Alexa-680 fluorescence imaging, whereas mice fed a regular diet showed high background fluorescence in the GIT (Figure 3 and Table 1). NIRFI showed no diet-associated background fluorescence (Table 1).

**Figure 4:**
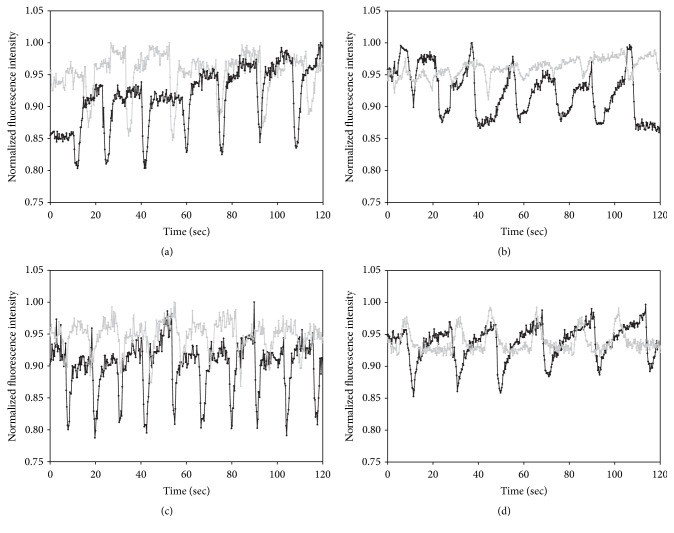
Representative fluorescent intensity profiles from NIRFI (black) and FRFI (gray) as a function of time in mice without (a) or with (b) skin pigmentation fed a special diet or in mice without (c) or with (d) skin pigmentation fed a regular diet.

**Figure 5:**
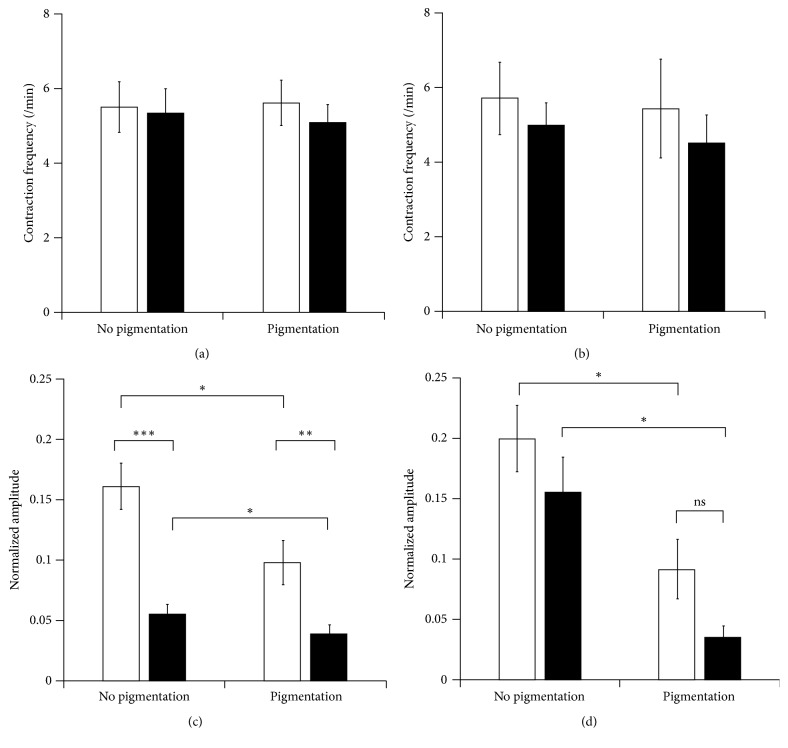
Quantification of lymphatic contraction frequency (a, b) and fluorescence signal amplitude in lymphatic contractions (c, d) in the skin with or without pigmentation, fed a regular ((a), (c); *n* = 9) or special ((b), (d); *n* = 7) diet after NIRFI (white) and FRFI (black). Data are shown as mean ± SEM. Paired and unpaired Student* T*-tests were performed in (c). Mann–Whitney and Wilcoxon tests were used to compare two unpaired or paired groups, respectively, in (d). ^*∗*^*P* < 0.05. ^*∗∗*^*P* < 0.01. ^*∗∗∗*^*P* < 0.001. NS, not significant (*P* = 0.1).

To reveal whether fluorescence imaging of lymphatic contraction frequency and fluorescent signal amplitude are affected by skin pigmentation and diet, we selected a ROI in the fluorescent collecting lymphatic vessel, normalized the profiles, and plotted them as a function of time. As shown in Figure 4, we observed dynamic changes of fluorescent intensities due to lymphatic contractile activities. We found no significant differences in lymphatic contraction frequency in all groups regardless of skin pigmentation or diet (Figures 5(a) and 5(b)). However, we observed reduced fluorescent signal amplitude of lymphatic contraction. In mice with and without pigmentation, fed a regular diet, we found significantly greater NIRF signal amplitude compared to Alexa-680 (Figure 5(c)). In addition, when mice were heavily pigmented, the NIR fluorescent and FR fluorescent signal amplitude were significantly reduced by 39% and 30%, respectively, as compared to those in nonpigmented mice (Figure 5(c)). As shown in Figure 5(d), we observed no difference in changes of the amplitude between ICG and Alexa-680 fluorescence signals in mice with and without pigmentation, fed an alfalfa-free diet. However, a significant reduction in the NIR and FR fluorescent amplitude was observed during skin pigmentation. The reduction of the amplitude in FRFI (77%) was greater than that in NIRFI (54%).

## 4. Discussion

In this study, we investigated whether lymphatic contraction frequency and signal amplitude change were affected in mice with depilation-induced skin pigmentation and diet-induced background fluorescence. FRFI and NIRFI have been widely used for in vivo animal imaging, since light from longer wavelength, for example, greater than 600 nm, can travel relatively long distances in vivo. However, fluorescence can be generated without administration of exogenous fluorophores. It has been reported that mice fed regular chow, which contains alfalfa, show strong diet-induced fluorescence in the stomach and intestines with a peak wavelength of about 680 nm [4]. Thus, nonspecific diet-induced background fluorescence may impair detection sensitivity by masking the signal from exogenous fluorescent dyes in the abdominal region during FRFI with light in the 600–700 nm wavelength. As a result, alfalfa-free diet has been used in animals to reduce diet-induced abdominal fluorescence to improve imaging performance. Our data showed significantly greater diet-induced background fluorescence in mice fed regular diet as compared to alfalfa-free diet, regardless of skin pigmentation, indicating diet-induced background fluorescence in mice with pigmented skin may further deteriorate imaging quality (Table 1). However, note that fluorescent signal from lymphatic vessels away from the abdominal regions (e.g., vessels in the paw) may not be affected by diet-induced background fluorescence. Taken together, depilation-induced pigmentation may complicate fluorescence signal amplitude measurement in C57BL6 mice as shown in Figures 3–5.

Mice in the resting (telogen) phase showed little or no pigmentation (week 1 in Figure 1). However, after chemical depilation, new hair follicles started to develop and the pink skin began to darken in mice, which were in the growth (anagen) phase. As the anagen phase progresses, the skin continues to darken in color. It has been demonstrated that the melanin content is related to hair follicle cycle. Melanocytes in actively growing hair follicles produce more melanin (exclusively eumelanin in C57BL6 mice) during depilation-induced hair growth [13]. Pigmentation is heaviest in the late anagen; thus light can hardly penetrate to the dermis due to the high absorption for melanin (Week 3 in Figure 1). Melanin is one of the major sources of light absorption in biological tissues [14–17]. Melanin absorbs light from the visible spectrum, and the absorption of melanin decreases with increasing wavelength [14–17]. Thus, the presence of melanin and varying pigmentation can impair quantitative and qualitative in vivo optical imaging. Curtis et al. [3] showed the significantly reduced bioluminescent signal, which emits light from green to yellow wavelengths with the peak emission at 562 nm, through pigmented skin (about an average of 90% signal attenuation) as compared to nonpigmented skin of mice. It has been suggested that melanin presents low absorption in the NIR range and thus NIRFI may be used to overcome the limitation of signal attenuation in skin pigmentation. However, as demonstrated in our current study, we observed that changes of NIR fluorescent signal amplitude were significantly impacted by skin pigmentation (Figures 5(c) and 5(d)). NIR fluorescent signal amplitude was reduced by 39% and 54% in pigmented mice with regular and alfalfa-free diet, respectively. In addition, the network of inguinal afferent lymphatic vessels in the hindlimb was clearly observed in mice without skin pigmentation when hair follicles were at the later catagen and telogen and the melanin content was lower; however, those vessel structures could not be properly delineated in dark skin pigmented mice due to strong light absorption by melanin. We longitudinally imaged nude mice fed with regular murine diet without depilation using NIRFI and found similar amplitude as that in C57BL6 mice with no pigmentation. However, further study is needed to investigate how fluorescence lymphatic imaging is impacted in response to chemical depilation in mice of different strains, such as C3H/He and nude mice, as compared to C57BL6 mice, or with different gender and age.

In conclusion, diet-induced background fluorescence acquired using FRFI significantly impacts lymphatic measurement of the fluorescent signal amplitude, but not lymphatic contraction frequency, in the internodal collecting lymphatic vessels in mice fed a regular diet, and skin pigmentation further affects the amplitude. However, feeding mice with an alfalfa-free diet removes autofluorescence caused by diet and thus improves the amplitude. Although NIRFI shows no or minimal background fluorescence in mice on either a regular or alfalfa-free diet, skin pigmentation affects the amplitude of NIR fluorescent signal changes. Therefore, care should be taken to study longitudinal imaging of C57BL6 mice and when imaging groups of mice with different status of skin pigmentation in vivo.

## Figures and Tables

**Table 1:** Summary of fluorescent intensities (a.u.) in the stomach area in pigmented or nonpigmented mice fed a regular or alfalfa-free diet. Data are shown as mean ± SEM of *n* = 6 mice per group. ^*∗∗∗*^*P* < 0.001 versus mice fed alfalfa-free diet. Unpaired Student *t*-test was performed.

	Non-skin pigmented		Skin pigmented	
	Regular	Alfalfa-free	Regular	Alfalfa-free
ICG	1723 ± 44	1699 ± 43	1607 ± 28	1548 ± 15
Alexa680	6799 ± 786^*∗∗∗*^	1763 ± 45	1827 ± 47^*∗∗∗*^	1519 ± 9

## References

[1] Stenn K. S., Paus R. (2001). Controls of hair follicle cycling. *Physiological Reviews*.

[2] Alonso L., Fuchs E. (2006). The hair cycle. *Journal of Cell Science*.

[3] Curtis A., Calabro K., Galarneau J.-R., Bigio I. J., Krucker T. (2011). Temporal variations of skin pigmentation in C57Bl/6 mice affect optical bioluminescence quantitation. *Molecular Imaging and Biology*.

[4] Troy T., Jekic-McMullen D., Sambucetti L., Rice B. (2004). Quantitative comparison of the sensitivity of detection of fluorescent and bioluminescent reporters in animal models. *Molecular Imaging*.

[5] Inoue Y., Izawa K., Kiryu S., Tojo A., Ohtomo K. (2008). Diet and abdominal autofluorescence detected by in vivo fluorescence imaging of living mice. *Molecular Imaging*.

[6] Liao S., Cheng G., Conner D. A. (2011). Impaired lymphatic contraction associated with immunosuppression. *Proceedings of the National Academy of Sciences of the United States of America*.

[7] Kwon S., Sevick-Muraca E. M. (2007). Noninvasive quantitative imaging of lymph function in mice. *Lymphatic Research and Biology*.

[8] Kwon S., Sevick-Muraca E. M. (2010). Functional lymphatic imaging in tumor-bearing mice. *Journal of Immunological Methods*.

[9] Kwon S., Sevick-Muraca E. M. (2011). Mouse phenotyping with near-infrared fluorescence lymphatic imaging. *Biomedical Optics Express*.

[10] Proulx S. T., Luciani P., Derzsi S. (2010). Quantitative imaging of lymphatic function with liposomal indocyanine green. *Cancer Research*.

[11] Proulx S. T., Luciani P., Christiansen A. (2013). Use of a PEG-conjugated bright near-infrared dye for functional imaging of rerouting of tumor lymphatic drainage after sentinel lymph node metastasis. *Biomaterials*.

[12] Weiler M., Kassis T., Dixon J. B. (2012). Sensitivity analysis of near-infrared functional lymphatic imaging. *Journal of Biomedical Optics*.

[13] Slominski A., Wortsman J., Plonka P. M., Schallreuter K. U., Paus R., Tobin D. J. (2005). Hair follicle pigmentation. *Journal of Investigative Dermatology*.

[14] Jacques S. L. (2013). Optical properties of biological tissues: a review. *Physics in Medicine and Biology*.

[15] Sardar D. K., Mayo M. L., Glickman R. D. (2001). Optical characterization of melanin. *Journal of Biomedical Optics*.

[16] Kollias N., Baqer A. (1985). Spectroscopic characteristics of human melanin in vivo. *Journal of Investigative Dermatology*.

[17] Tseng S.-H., Bargo P., Durkin A., Kollias N. (2009). Chromophore concentrations, absorption and scattering properties of human skin in-vivo. *Optics Express*.

